# Radiometal-Based PET/MRI Contrast Agents for Sensing Tumor Extracellular pH

**DOI:** 10.3390/bios12020134

**Published:** 2022-02-20

**Authors:** Alyssa C. Pollard, Jorge de la Cerda, F. William Schuler, Tyler R. Pollard, Aikaterini Kotrotsou, Federica Pisaneschi, Mark D. Pagel

**Affiliations:** 1 Department of Chemistry, Rice University, Houston, TX 77005, USA; acp4@rice.edu; 2 Department of Cancer Systems Imaging, MD Anderson Cancer Center, Houston, TX 77054, USA; jorge.delacerda@mdanderson.org (J.d.l.C.); fschuler@mdanderson.org (F.W.S.); aikaterini.kotrotsou@gmail.com (A.K.); fpisaneschi@mdanderson.org (F.P.); 3 Department of Chemistry and Biochemistry, Georgia Institute of Technology, Atlanta, GA 30332, USA; polko987@outlook.com

**Keywords:** PET/MRI, contrast agents, pH, cancer, gallium-68, copper-64

## Abstract

Acidosis is a useful biomarker for tumor diagnoses and for evaluating early response to anti-cancer treatments. Despite these useful applications, there are few methods for non-invasively measuring tumor extracellular pH, and none are routinely used in clinics. Responsive MRI contrast agents have been developed, and they undergo a change in MRI signal with pH. However, these signal changes are concentration-dependent, and it is difficult to accurately measure the concentration of an MRI contrast agent in vivo. PET/MRI provides a unique opportunity to overcome this concentration dependence issue by using the PET component to report on the concentration of the pH-responsive MRI agent. Herein, we synthesized PET/MRI co-agents based on the design of a pH-dependent MRI agent, and we have correlated pH with the r_1_ relaxivity of the MRI co-agent. We have also developed a procedure that uses PET radioactivity measurements and MRI R_1_ relaxation rate measurements to determine the r_1_ relaxivity of the MRI co-agent, which can then be used to estimate pH. This simultaneous PET/MRI procedure accurately measured pH in solution, with a precision that depended on the concentration of the MRI co-agent. We used our procedure to measure extracellular pH in a subcutaneous flank model of MIA PaCa-2 pancreatic cancer. Although the PET co-agents were stable in serum, post-imaging studies showed evidence that the PET co-agents were degraded in vivo. These results showed that tumor acidosis can be evaluated with simultaneous PET/MRI, although improvements are needed to more precisely measure MRI R_1_ relaxation rates, and ensure the in vivo stability of the agents.

## 1. Introduction

**Figure 1:**
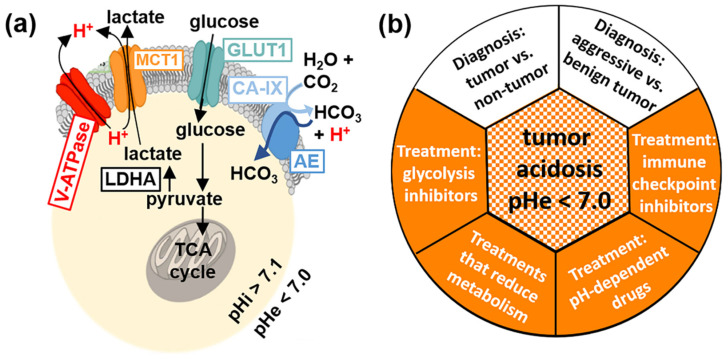
Tumor acidosis. (**a**) The Warburg effect leads to an acidic tumor microenvironment. GLUT1 and other glucose transporters shuttle glucose into the cell, which is metabolized into pyruvate. The pyruvate is then converted into lactate by lactate dehydrogenase (LDHA), and this upregulated glycolysis pathway in tumor cells is known as the Warburg effect. The lactate is then secreted out of tumor cells by MCT1 and other monocarboxylate transporters, along with excess intracellular protons secreted via V-ATPase (red H^+^, left side). Furthermore, carbonic anhydrase IX (CA-IX) produces bicarbonate that is internalized into cells to neutralize intracellular acidosis caused by the Warburg effect, and the bicarbonate production contributes to extracellular acidification (red H^+^, right side). These processes lead to an acidic extracellular pH (pHe) relative to intracellular pH (pHi). (**b**) Evaluations of tumor acidosis can have a central role in diagnostic applications (white) and evaluations of anti-cancer treatments (orange).

Cancer cells undergo many biochemical processes that distinguish them from normal cells. One of these processes is upregulated aerobic glycolysis, which has been termed the Warburg effect (Figure 1a) [1]. Due to this effect, cancer cells upregulate the conversion of glucose into lactate through glycolysis, even in the presence of adequate oxygen [1,2]. The Warburg effect is atypical for normal cells because aerobic glycolysis is an inefficient pathway to synthesize adenosine 5’-triphosphate (ATP). While there is still debate regarding the selective pressures for tumors to rely on the Warburg effect, the production of lactate is an effective method to quickly produce carbon building blocks, which aid in rapid cancer cell division [2]. Once lactate is produced, it is then secreted out of the tumor cells, leading to a buildup of extracellular lactic acid, and an acidic extracellular pH (pHe) (<7.0), compared to normal tissues [2,3].

The evaluation of tumor acidosis has many diagnostic applications (Figure 1b). Tumor acidosis is related to tumor growth rate [4], invasion [5], and metastasis [6], so quantitatively imaging pHe can improve the diagnosis of aggressive vs. benign tumors. Measuring pH can also differentiate acidic tumors with pHe < 7.0 from mild inflammation that typically has pHe 7.1–7.4 [7], and infections that are typically pHe-neutral [8]. Moreover, monitoring changes in pHe can evaluate the early response to certain drug therapies that directly reduce tumor glycolysis, or that more generally reduce tumor metabolism [9,10]. Evaluations of tumor pHe can also be used to predict and monitor the effect of pH-dependent drugs and antibodies [11,12]. Tumor acidosis contributes to immune escape, and therefore, measuring tumor pHe can aid in predicting response to immunotherapies before expensive treatments are started [13].

While many research studies have explored methods to measure in vivo tumor acidosis through molecular imaging, it is currently not a practice adopted in the clinic [14]. In particular, magnetic resonance imaging (MRI) has been investigated for measuring in vivo tumor pHe. T_1_ agents have an r_1_ relaxivity that indicates the strength of the agent to increase the R_1_ relaxation rate of water, and generate T_1_-weighted MR image contrast (Equations (1) and (2)) [15]. Some T_1_ contrast agents have been designed to have a pH-dependent r_1_ relaxivity, so that the measured ΔR_1_ may be used to determine pH [16,17]. However, the change in MR image contrast caused by these agents also depends on the agent’s concentration ([CA]) in the tissue (Equation (3)):

R_1_ = 1/T_1_ (1)

ΔR_1_ = R_1,with agent_ − R_1,without agent_ (2)

ΔR_1_ = r_1_ [CA] (3)

The concentration of an MRI contrast agent is variable between different tissues, and even within the same tissue. For this reason, using a single pH-responsive MRI contrast agent is impractical for measuring in vivo tumor pHe [18].

Many researchers have devised unique ways to address this concentration dependence, by using ratiometric approaches that involve pH-dependent and pH-independent imaging agents [18]. One solution is to harness the power of simultaneous PET/MRI [19], where the radioactivity measured by positron emission tomography (PET) corresponds to the concentration of the agent, and is also independent of pH [20]. This PET-based concentration measurement can then be used in a ratiometric approach with the pH- and concentration-dependent MR image contrast that is affected by the MRI agent, resulting in an estimated pH value.

In this work, a pair of contrast agents have been synthesized and consist of a pH-responsive MRI co-agent and a pH-independent PET co-agent. These two agents were designed to have identical pharmacokinetics, so that a dual injection of the two agents at a known ratio can quantitatively measure pH. The r_1_ relaxivity of the MRI co-agent has been reported to relate to pH, because the protonation of the sulfonamide leads to the dissociation of the arm from the gadolinium (Gd) core, increasing the R_1_ relaxation rate of the surrounding water [21]. With a known ratio of MRI co-agent to PET co-agent, the radioactivity detected by the PET detector can be translated into the concentration of the MRI co-agent. Therefore, with this known concentration and the R_1_ relaxation rate, the r_1_ relaxivity can be calculated, and pH can be determined using a calibration curve of pH versus r_1_ relaxivity for the MRI co-agent. This approach has been explored previously with a different pair of PET/MRI co-agents than the agents used in our study, but was not tested with a clinically relevant magnetic field strength, and in vivo studies were not performed [20].

## 2. Materials and Methods

### 2.1. General Chemistry and Radiochemistry Methods

All solvents and reagents were purchased from commercial sources and used as received. Water was deionized using a Milli-Q integral water purification system (MilliporeSigma, Burlington, MA, USA). The pH values of the samples were recorded using a SevenCompact S221 benchtop pH/ion meter (Mettler Toledo, Columbus, OH, USA). All reactions were performed with oven-dried glassware under nitrogen, unless otherwise noted. Thin layer chromatography (TLC) was performed using aluminum-backed plates pre-coated with silica gel 60 matrix with fluorescent indicator F_254_ and 0.2 mm layer thickness. Flash column chromatography was performed using an Isolera One automated flash chromatography system (Biotage, Uppsala, Sweden). ^1^H NMR spectra were performed using a 500 or 600 MHz Bruker NMR spectrometer (Bruker, Billerica, MA, USA). Chemical shifts (δ) are reported in ppm. Ultra-performance liquid chromatography-mass spectrometry/mass spectrometry (UPLC-MS/MS) was performed using a Waters Xevo TQD IVD with Acquity UPLC, using an Acquity UPLC BEH C18 1.7 μm column with 2.1 × 50 mm dimensions (Waters, Milford, MA, USA). The concentration of the MRI co-agent was confirmed by measuring Gd concentration using a PerkinElmer NexION 300 inductively coupled plasma-mass spectrometer (ICP-MS) running Syngistix software (PerkinElmer, Waltham, MA, USA). The acquisition mode included three replicates averaged to give ^157^Gd concentrations. The dwell time was 50 ms with 18 L/min main argon flow, 1.2 L/min auxiliary argon flow, 0.97 L/min optimized nebulizer argon flow, 1600 W RF power, 0.2 mL/min sample flow, and KED cell mode with 1.2 mL/min helium flow.

Preparative HPLC (prep HPLC) was performed on an Agilent 1260 Infinity II (Agilent Technologies, Santa Clara, CA, USA) with a Luna^®^ C18 5 μm column, with 21.2 × 250 mm dimensions (Phenomenex, Torrance, CA, USA). RadioHPLC was performed on an analytical Agilent 1260 Infinity II Series (Agilent Technologies, Santa Clara, CA, USA) with a XBridge C18 3.5 μm column with 4.6 × 250 mm dimensions using a Flow-RAM radioHPLC detector (LabLogic Systems Ltd., Brandon, FL, USA), for compound identification and quality control. For metabolism studies, a more sensitive system was warranted, so an analytical Agilent 1100 Series system (Agilent Technologies, Santa Clara, CA, USA) was used with an Econosil 10 μm column with 4.6 × 250 mm dimensions (Alltech, Nicholasville, KY, USA). Counts were detected using a Bioscan Model 106 detector (Bioscan, Inc., Poway, CA, USA) interfaced with the HPLC using an Agilent Interface 35900E (Agilent Technologies, Santa Clara, CA, USA). Purification with a C18 cartridge was performed using a light C18 Sep Pak^®^ cartridge (Waters, Milford, MA, USA) prewashed with ethanol (3 mL), and then water (6 mL). HPLC was performed using one of the following solvent systems: 0.1% TFA in water (solvent A) and 0.1% TFA in acetonitrile (solvent B); or 0.05% formic acid in water (solvent C) and 0.05% formic acid in acetonitrile (solvent D). For prep HPLC, the following method was used: 5% solvent B (in solvent A) to 95% solvent B (in solvent A) over 34 min, then hold at 95% solvent B (in solvent A) for 3 min with a 20 mL/min flow rate. For analytical HPLC, the following method was used: 5% solvent D (in solvent C) to 95% solvent D (in solvent C) over 15 min with a 1 mL/min flow rate.

[^64^Cu]CuCl_2_ (t_1/2_ = 12.7 h, β^+^ % = 18%) was produced from a 16 MeV proton/deuteron GE PETtrace 10 cyclotron (GE Healthcare, Chicago, IL, USA) using an EDS/PTS solid target station (Comecer S.p.A., Castel Bolognese, Italy) in the Cyclotron Radiochemistry Facility at the MD Anderson Cancer Center. [^68^Ga]GaCl_3_ (t_1/2_ = 68 min, β^+^ % = 89%) was produced from a 1.85 GBq capacity GalliaPharm^® 68^Ge/^68^Ga Radionuclide Generator (Eckert & Ziegler Radiopharma GmbH, Berlin, Germany) in the Cyclotron Radiochemistry Facility at the MD Anderson Cancer Center.

### 2.2. Synthesis of MRI Co-Agent

Compound **1**: Cyclen (300 mg, 1.74 mmol) was dissolved in DMA (3.6 mL) and cooled to −20 °C and NaOAc (471 mg, 5.74 mmol) was added. *Tert*-butylbromoacetate (0.85 mL, 5.74 mmol) was dissolved in DMA (1.2 mL) and added dropwise to the cyclen solution over 30 min while maintaining the temperature. The reaction was then returned to room temperature and stirred for 24 h. The reaction mixture was then poured into water to give a clear solution, and KHCO_3_ was added portionwise until a precipitate formed. The precipitate was filtered, washed adequately with water, and lyophilized to dryness to give a white solid (838 mg, 81% yield). ^1^H NMR (600 MHz, CDCl_3_): δ = 9.99 (br s, 1H), 3.37 (s, 4H), 3.29 (s, 2H), 3.09 (s, 4H), 2.94–2.89 (m, 8H), 2.86 (s, 4H), 1.46 (s, 18H), 1.45 (s, 9H). LRMS (ESI): *m*/*z* calculated for C_26_H_51_N_4_O_6_ [M + H]^+^ requires 515.38; found 515.14.

Compound **2**: 4-Methoxybenzenesulfonyl chloride (200 mg, 0.968 mmol) and 2-bromoethylamine hydrobromide (228 mg, 1.11 mmol) were suspended in DCM (3.3 mL) and cooled to 0 °C. Triethylamine (0.32 mL, 2.32 mmol) was added dropwise over 10 min, and the reaction mixture was stirred for 1 h. The reaction was diluted with DCM and washed with 1 M HCl two times followed by brine. The organic layer was dried over MgSO_4_ and evaporated under reduced pressure. The resulting oil was purified by column chromatography over silica gel eluting with a gradient of 12% ethyl acetate to 100% ethyl acetate in hexanes over 10 column volumes to afford the title compound as a white solid (220 mg, 77% yield). ^1^H NMR (500 MHz, CDCl_3_): δ = 7.80 (d, *J* = 9.0 Hz, 2H), 6.99 (d, *J* = 9.0 Hz, 2H), 4.94 (br s, 1H), 3.88 (s, 3H), 3.43–3.32 (m, 4H). LRMS (ESI): *m*/*z* calculated for C_9_H_13_BrNO_3_S [M + H]^+^ requires 293.97; found 294.19.

Compound **3**: Compound **1** (118 mg, 0.198 mmol) was dissolved in ACN (2.3 mL), and K_2_CO_3_ (82 mg, 0.594 mmol) was added. Compound **2** (70 mg, 0.238 mmol) was dissolved in ACN (0.59 mL). The reaction was cooled to 0 °C, and the solution of compound **2** was added dropwise. The reaction mixture was stirred overnight at room temperature, then K_2_CO_3_ was removed via vacuum filtration. The filtrate was evaporated under reduced pressure, and the resulting oil was purified by prep HPLC and lyophilized to afford the title compound as a white solid (141 mg, 98% yield). ^1^H NMR (600 MHz, CDCl_3_): δ = 7.78 (d, *J* = 8.8 Hz, 2H), 6.97 (d, *J* = 8.9 Hz, 2H), 3.86 (s, 3H), 3.80–2.67 (m, 27H), 1.49 (s, 9H), 1.45 (s, 18H). LRMS (ESI): *m*/*z* calculated for C_35_H_62_N_5_O_9_S [M + H]^+^ requires 728.42; found 728.66.

Compound **4**: Compound **3** (1.447 g, 1.988 mmol) was dissolved in concentrated HCl (10 mL) and stirred for 3 h at room temperature. The solution was then evaporated under reduced pressure, redissolved in water, and filtered via a 0.2 μm syringe filter. The filtrate was lyophilized and used without further purification to afford the title compound as white solid (1.078 g, 97% yield). ^1^H NMR (600 MHz, D_2_O) δ = 7.76 (d, *J* = 9.0 Hz, 2H), 7.09 (d, *J* = 9.0 Hz, 2H), 3.83 (s, 3H), 3.72–3.50 (m, 6H), 3.32–3.03 (m, 20H). LRMS (ESI): *m*/*z* calculated for C_23_H_38_N_5_O_9_S [M + H]^+^ requires 560.23; found 560.03.

Compound **5**: Compound **4** (400 mg, 0.715 mmol) and GdCl_3_ (226 mg, 0.858 mmol) were dissolved in 2 mL H_2_O, and the pH was raised to 5–6 with the addition of 1 M NaOH. The reaction mixture was heated to 90 °C and stirred for 12 h. The pH was then raised to 11–12, and the solution was centrifuged for 8 min at 4000 rpm. The supernatant was removed, and the pellet was washed with water two more times, centrifuging and removing the supernatant between each wash. The supernatants were combined and checked for free gadolinium using arsenazo III dye [22]. The solution was then neutralized with 1 M HCl and lyophilized. The resulting solid was dissolved in 95% DCM in MeOH and filtered via vacuum filtration to remove salt byproducts. The filtrate was evaporated under reduced pressure, redissolved in water, filtered via a 0.2 μm syringe filter, and lyophilized to afford the title compound as a white solid (510 mg, quant.). LRMS (ESI): *m*/*z* calculated for C_23_H_32_GdN_5_O_9_ [M - H]^-^ requires 712.12; found 712.36.

### 2.3. Radiosynthesis of PET Co-Agents

Compound **6**: 1.51 mCi (about 700 μL) of [^68^Ga]GaCl_3_ in 0.05 M HCl was added to 300 μL of 1 M HEPES buffer, pH = 4.8. Then 20 μL of compound **4** (2 mg/mL solution in water) was added, and the reaction was heated to 85 °C for 10 min. Reaction completion was confirmed with radioHPLC. The reaction mixture was loaded onto a pre-conditioned C18 cartridge and eluted with 1 mL of a 1:1 PBS:ethanol mixture in 100 μL fractions to concentrate the solution. A non-decay-corrected radiochemical yield of 51% was obtained (n = 9) with a radiochemical purity of 98%.

Compound **7**: 4.74 mCi (about 3 μL) of cyclotron produced [^64^Cu]CuCl_2_ in 0.1 M HCl was added to 100 μL of 0.1 M NaOAc buffer, pH = 5.6. 20 μL of compound **4** (2 mg/mL solution in water) was added, and the reaction was heated to 37 °C for 30 min. Reaction completion was confirmed with radioHPLC. The reaction mixture was loaded onto a pre-conditioned C18 cartridge and with 1 mL of a 1:1 PBS:ethanol mixture in 100 μL fractions to concentrate the solution. A non-decay-corrected radiochemical yield of 81% (n = 1) was obtained with a radiochemical purity of 99%.

### 2.4. pH-Relaxivity Calibration in Solution

A matrix of samples containing the MRI co-agent in 10 mM HEPES were made at concentrations of 0, 0.1, 0.5, 1.2, and 2 mM, and pH values from 4–9.8, with approximately 0.12 pH unit steps, for a total of 235 samples. Each sample was prepared in a PCR tube of 200 μL volume. Concentrations were confirmed using ICP-MS, and pH values were confirmed using a calibrated pH meter. Samples were arranged in a small box filled with 2% agarose designed to hold 30 samples and fit into a Bruker 72 mm MRI coil. The temperature of the samples was maintained at 37 °C with warm air, as validated with the NMR spectroscopy of ethylene glycol samples in an identical agarose-filled box and instrument setup [23].

The samples were imaged using a 7T MR scanner with a 30 cm horizontal bore equipped with 20 cm fixed gradients and Avance HD architecture, using a 72 mm transceiver volume coil (Bruker Biospin, Billerica, MA, USA). The following two MRI acquisition methods were used for the imaging experiment: (1) 2D coronal rapid acquisition with relaxation enhancement with variable repetition time (RAREVTR) (TE = 21.68 ms, 12 TR = 300, 583.697, 897.052, 1246.995, 1643.217, 2099.848, 2638.683, 3295.960, 4138.860, 5315.362, 7278.213, 15,000 ms, RARE factor = 8, echo spacing = 5.420 ms, 16 dummy scans, 1 average, 1 repetition, 1 coronal slice, slice thickness = 1 mm, 100 × 100 mm FOV, 128 × 128 matrix, 0.781 × 0.781 mm resolution); (2) 2D RAREVTR (TE = 21.68 ms, 12 TR = 150, 244.528, 348.933, 465.522, 597.522, 749. 633, 929.107, 1147.994, 1428.622, 1820.126, 2472.524, 5000 ms, RARE factor = 8, echo spacing = 5.420 ms, 16 dummy scans, 1 average, 1 repetition, 1 coronal slice, slice thickness = 1 mm, 100 × 100 mm FOV, 128 × 128 matrix, 0.781 × 0.781 mm resolution). MR images were reconstructed, and T_1_ times were calculated using ParaVision 6.0.1 (Bruker Biospin, Billerica, MA, USA).

T_1_ times from the first acquisition method were used for samples with 0, 0.1, and 0.5 mM concentrations, and T_1_ times from the second acquisition method were used for the samples with 1.2 and 2 mM concentrations. T_1_ times were then converted to R_1_ relaxation rates. These rates were plotted versus concentration, and the slope of the line was used to determine r_1_ relaxivity for each pH value. The r_1_ relaxivity was then plotted versus pH values to produce a pH-relaxivity calibration curve. These data were fit to a modified Henderson–Hasselbach equation (Equation (4)) using Matlab R2021b (MathWorks, Natick, MA, USA). This equation was used to convert relaxivity to pH in subsequent PET/MRI experiments:

(4) $$\mathrm{pH}=\mathrm{pKa}+\log\left(\frac{r_{1}-a}{b-r_{1}}\right),$$

where pKa is the pKa of the sulfonamide arm on the MRI co-agent, r_1_ is the relaxivity of the measured sample, a is the relaxivity when the compound is completely protonated, and b is the relaxivity when the compound is completely deprotonated.

### 2.5. Imaging pH in Solution with PET/MRI

Samples were prepared in a 3 mL volume of 10 mM HEPES containing concentrations of 0.05, 0.1, 0.25, 0.5, and 0.8 mM of the MRI co-agent at 7 pH values ranging from 6.2–7.4 pH units. The concentrations of the MRI co-agent were confirmed using ICP-MS. Each sample was spiked with 10–45 μCi of the ^68^Ga PET co-agent. The amount of PET co-agent was measured using a CRC-15R dose calibrator (Capintec, Inc., Florham Park, NJ, USA), and decay-corrected to the start of the PET/MRI scan. The samples were placed inside a 15 mL conical tube filled with 2% agarose.

All simultaneous PET/MRI scans were performed on the same 7T MRI instrument with a NuPET™ insert (Cubresa, Inc., Winnipeg, MB, USA) and a 35 mm MRI transceiver volume coil (Bruker Biospin, Billerica, MA, USA). For PET/MRI studies in solution, a simultaneous 30-min PET scan was obtained while also performing the two MRI acquisitions described in Section 2.4, except that an FOV of 38.4 × 38.4 mm was used for a resolution of 0.300 × 0.300 mm. MR image reconstructions and T_1_ times were calculated using ParaVision 6.0.1 (Bruker, Billerica, MA, USA). Random and decay corrections for PET images were applied through the Cubresa software. PET reconstructions were performed using an OSMAPOSL algorithm with 8 iterations and 4 subsets, and PET images were quantified using a Quantification Calibration Factor for ^68^Ga or ^64^Cu. PET VOIs and representative images were generated and analyzed using VivoQuant (Invicro LLC, Needham, MA, USA).

A VOI was drawn around the tube in the PET image, and the total amount of radioactivity in each sample was determined via the NuPET™ software. This value was converted to the concentration of the MRI co-agent using the known ratio of the PET-to-MRI co-agents in the sample, which simulated the process used to determine the concentration of the MRI co-agent during in vivo studies. The T_1_ relaxation times were converted to R_1_ relaxation rates. The value of ΔR_1_ was calculated for each sample using the R_1_ relaxation rate at 0 mM of MRI co-agent during the pH-relaxivity calibration curve experiments (Section 2.4). This value was divided by the calculated concentration of the MRI co-agent, which determined the r_1_ relaxivity of the sample. Finally, this value for r_1_ relaxivity was converted to a pH value for each sample using the pH-relaxivity calibration curve (Equation (4)). This value was compared to the pH of the sample measured with a calibrated pH meter. Statistical analysis was performed using Microsoft Excel (Microsoft, Corp., Redmond, WA, USA).

### 2.6. Mouse Model

All experiments with mice were approved by the Institutional Animal Care and Use Committee of the University of Texas MD Anderson Cancer Center (MDACC) and Rice University. MIA PaCa-2 pancreatic tumor cells were grown in Dulbecco’s modified Eagle media (10-017-CV, Corning, Inc., Corning, NY, USA), with 10% FBS in a T75 flask. Female athymic nude mice were obtained from the MDACC Experimental Radiation Oncology mouse colony. Subcutaneous tumor models were generated by the subcutaneous injection of 50 µL of phosphate buffered saline, containing 1.5 million MIA PaCa-2 cells and 50 µL of Matrigel (Corning, Inc., Corning, NY, USA). The tumors were allowed to grow to 5 mm in diameter within 6 weeks.

### 2.7. Imaging pH In Vivo with PET/MRI

Two mice were used to test the ^64^Cu PET co-agent, and one mouse was used to test the ^68^Ga PET co-agent during in vivo PET/MRI studies. Each mouse was placed on a sled fitted with a nose cone, allowing the mouse to be anesthetized with 2% isoflurane, using oxygen as a carrier throughout the imaging experiment. A heated water pad under the mouse and temperature-controlled warm air (SA Instruments, Stony Brook, NY, USA) were used to maintain mouse temperature at 37 °C. The breathing rate was monitored throughout the experiment. Each mouse was catheterized via tail vein, and a 125 μL line was used to connect the needle to the syringe located outside of the scanner. This line was filled with a 25 μL lead of saline, followed by a 250 μL injection volume of the PET/MRI co-agents (which completely filled the line and partially filled the syringe). More specifically, 120 μCi (~80 μL) of the PET co-agent and 170 μL of a 30 mM solution of the MRI co-agent were injected in a slow bolus over 1 min, starting 1 min after initiating the DCE MRI scan (to acquire 1 min of baseline images prior to injection; see acquisition method 4 described in the next paragraph). Then, 125 μL of saline was then used to completely flush the co-agents out of the line and into the mouse, so the mice received a total injection volume of 275 μL. The concentration of the injected MRI co-agent was confirmed using ICP-MS. The injected dose of the PET co-agent was calculated by measuring the pre- and post-injection activity in the syringe using a CRC-15R dose calibrator (Capintec, Inc., Florham Park, NJ, USA), and decay-corrected to the start of the scan. It was assumed that the percentage loss of the PET and MRI co-agents in the syringe was equal.

The following MRI acquisitions were obtained for all in vivo imaging sessions: (1) a localizer image to ensure proper placement of the mouse in the PET/MRI system; (2) pre-injection 2D coronal RARE (TE = 43.99 ms, TR = 1200 ms, RARE factor = 8, echo spacing = 5.499 ms, 2 dummy scans, 2 averages, 1 repetition, 9 slices, slice thickness = 1 mm, 58.8 × 38.4 mm FOV, 196 × 128 matrix, 0.300 × 0.300 mm in-plane resolution); (3) 2D coronal RAREVTR (TE = 27.12 ms, 6 TR = 500, 650, 1000, 1500, 2500, 5000 ms, RARE factor = 8, echo spacing = 6.78 ms, 2 dummy scans, 2 averages, 1 repetition, 9 slices, slice thickness = 1 mm, 58.8 × 38.4 mm FOV, 196 × 128 matrix, 0.300 × 0.300 mm); (4) 2D coronal dynamic contrast enhancement fast low angle shot (DCE FLASH) (TE = 2.03 ms, TR = 59.462 ms, flip angle = 35°, 1 average, 130 repetitions, 9 slices, slice thickness = 1 mm, 58.8 × 38.4 mm FOV, 196 × 128 matrix, 0.300 × 0.300 mm); (5) post-injection 2D coronal RARE (TE = 43.99 ms, TR = 1200 ms, RARE factor = 8, echo spacing = 5.499 ms, 2 dummy scans, 2 averages, 1 repetition, 9 slices, slice thickness = 1 mm, 58.8 × 38.4 mm FOV, 196 × 128 matrix, 0.300 × 0.300 mm).

The dynamic PET scan was obtained simultaneously with the DCE MRI scan. Random and decay corrections for PET images were applied through the NuPET™ software. For the dynamic PET analysis, data were binned into 2-min time frames over the course of the 16-min dynamic scan. To produce static images, data were binned over the course of the entire 16-min PET scan. PET reconstructions were performed using an Ordered Subset Maximum a Posteriori One Step Late (OMAPOSL) algorithm with 8 iterations and 4 subsets, and PET images were quantified using a Quantification Calibration Factor for ^68^Ga or ^64^Cu. MRI reconstructions were performed using ParaVision 6.0.1 (Bruker Biospin, Billerica, MA, USA). PET/MR image registration was manually performed using the pre-injection MR image and VivoQuant (Invicro, LLC, Needham, MA, USA). PET VOIs and representative PET/MR images were also generated and analyzed using VivoQuant. Injected doses were used to calculate the percentage of injected dose per cubic centimeter of tissue (%ID/cc). Errors in the averaged %ID/cc were reported as standard deviation. MRI RAREVTR and DCE data were analyzed using Matlab R2021b (MathWorks, Natick, MA, USA).

After the reconstruction and registration of the PET/MR images, a VOI was drawn over the tumor using the anatomical MR image for reference. The total amount of radioactivity in the tumor was determined via the imaging software for each 2-min dynamic PET scan, which was decay-corrected to the start of the scan. This value was converted to the concentration of the MRI co-agent in the tumor for each time frame, using the known ratio of the PET-to-MRI co-agents in the injection volume and the physical tumor volume. The RAREVTR and DCE MR images were analyzed with Matlab to determine the relaxation rate for each 7.6 s MRI frame. The averages of the pre-injection frames of the DCE scan were subtracted from each post-injection frame, to determine ΔR_1_ for each time frame. ΔR_1_ was divided by the calculated MRI co-agent concentration for each 2-min time frame to determine relaxivity. Once the relaxivity reached a steady value, these relaxivity values were averaged, and the average value was used, with the modified Henderson–Hasselbach equation (Equation (4)), to determine tumor pHe.

### 2.8. Stability and Metabolism Studies of PET Co-Agents

The ^68^Ga PET co-agent (~100 μCi, ~100 μL) or ^64^Cu PET co-agent (~100 μCi, ~20 μL) was incubated in 5× volume of human serum or PBS at 37 °C. Aliquots were removed every 20 min and subjected to radioHPLC to determine the stability of the PET co-agent. For the ^64^Cu co-agent, an incubation in human serum and a PBS at 37 °C were also performed in the presence of a 100 mM concentration of the MRI co-agent. Aliquots were again removed every 20 min and subjected to radioHPLC to determine the stability of the ^64^Cu PET co-agent.

Two tumor-bearing mice (one for testing the ^68^Ga PET co-agent, and one for testing the ^64^Cu PET co-agent) were anesthetized with 2% isoflurane using oxygen as a carrier and injected i.v., with approximately 120 μCi (~80 μL) of the PET co-agent, and 170 μL of a 30 mM solution of the MRI co-agent. Mice remained anesthetized for twenty min, then urine was extracted, and the mice were sacrificed via cervical dislocation. All urine samples were treated with 2 × their volume of acetonitrile. The samples were mixed thoroughly and subjected to centrifugation for 10 min at 4000 rpm. The acetonitrile layer was removed and injected into the radioHPLC to determine the composition of the PET co-agent.

## 3. Results

### 3.1. Synthesis of PET/MRI Co-Agents

**Scheme 1:**
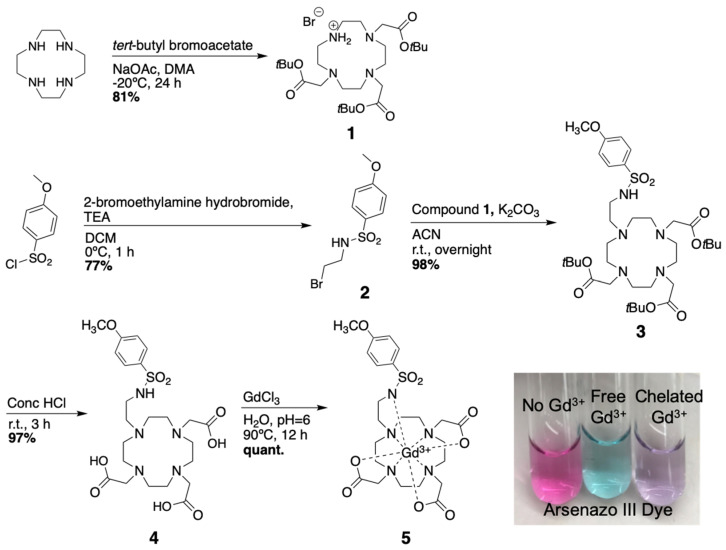
Synthesis of the pH responsive MRI co-agent.

**Scheme 2:**
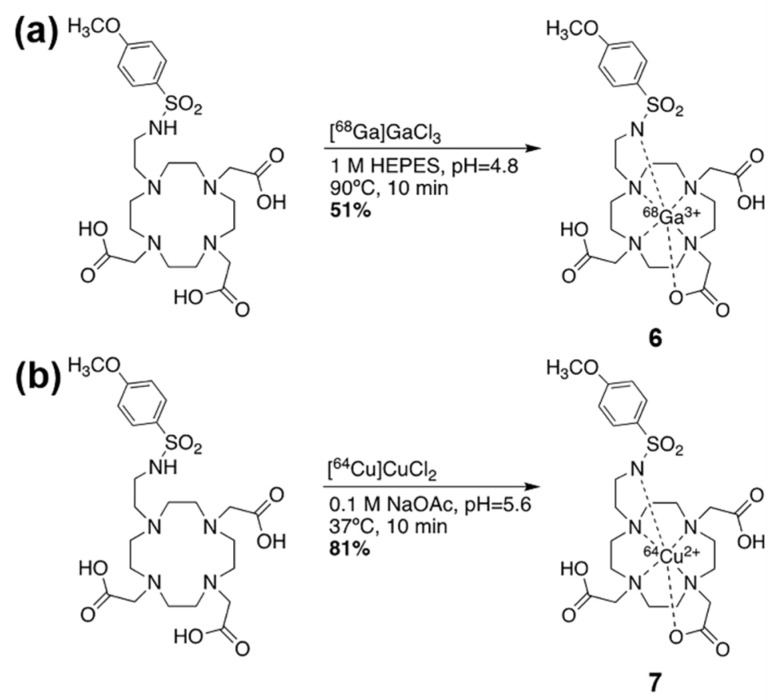
Radiosynthesis of radiometal-based PET co-agents. (**a**) The chelation of gallium-68 was performed with 51% yield. (**b**) The chelation of copper-64 was performed with 81% yield.

The MRI co-agent was synthesized with an overall yield of 59% over 5 steps (Scheme 1). The intermediates and final product were confirmed with NMR spectroscopy (Figures S1–S4) and LRMS (Figures S5–S9). Because free Gd metal could drastically affect relaxivity values, and is also as a known toxin [24], the absence of free Gd was confirmed at the end of each chelation using arsenazo III, which turns blue in the presence of the free metal ion (Scheme 1 inset) [22]. The ^68^Ga PET co-agent was synthesized with a radiochemical yield of 51% and a radiochemical purity of 98% (Scheme 2a). The ^64^Cu PET co-agent was synthesized with a radiochemical yield of 81% and a radiochemical purity of 99% (Scheme 2b).

### 3.2. pH-Relaxivity Calibration

**Figure 2:**
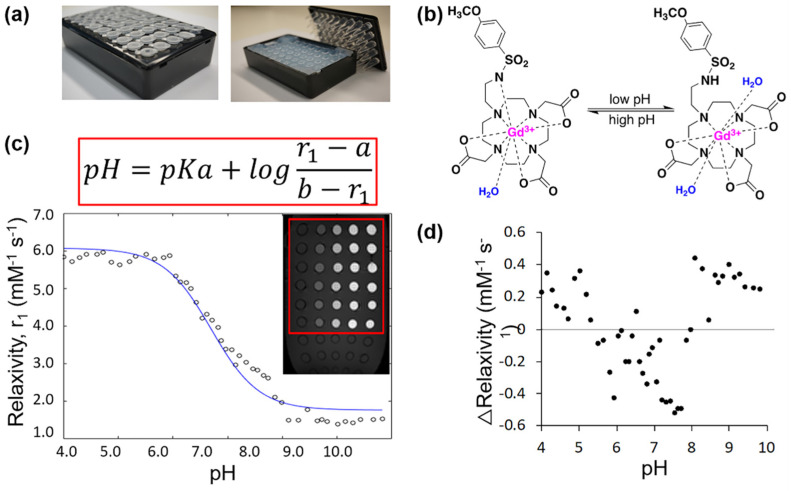
The MRI co-agent displayed pH-dependent relaxivities in a 7T MR scanner. (**a**) A phantom holder for pH-relaxivity calibration curve studies contained 2% agarose to reduce B0 and B1 inhomogeneities. (**b**) The pH responsive MRI co-agent undergoes protonation of its sulfonamide arm at low pH values, which allows for an additional water to occupy the inner sphere and interact with the gadolinium core. This leads to an increase in the relaxation rate of bulk water. At high pH values, this arm is deprotonated and bound to gadolinium, which leads to a decrease in the relaxation rate of bulk water. (**c**) Relaxation rates were calculated from measured T_1_ times of samples at various pH values and concentrations of the MRI co-agent. Relaxivities were calculated from the relaxation rates and concentrations, and the pH-relaxivity curve was fit to a modified Henderson–Hasselbach equation, where a is the r_1_ relaxivity at complete agent protonation and b is the r_1_ relaxivity at complete agent deprotonation. The image insert is a representative MR image of one sample box, where samples with increasingly higher concentrations of MRI co-agent appear increasing brighter towards the right side of the image. (**d**) The residuals of the fit of the pH-relaxivity curve demonstrated the good fit of the data.

Two different MRI acquisition methods were used for the creation of the calibration curve, because higher relaxation rates required shorter experimental TR times to obtain accurate T_1_ measurements. The pH-relaxivity calibration curve was fit using a modified Henderson–Hasselbach equation (Equation (4)) and the known pKa of 6.7 of the MRI co-agent [21]. The constants a and b were calculated to be 6.07 mM^−1^ s^−1^ and 1.77 mM^−1^ s^−1^, respectively (Figure 2). Residuals demonstrated the good fit of the data.

### 3.3. Imaging pH in Solution with PET/MRI

**Figure 3:**
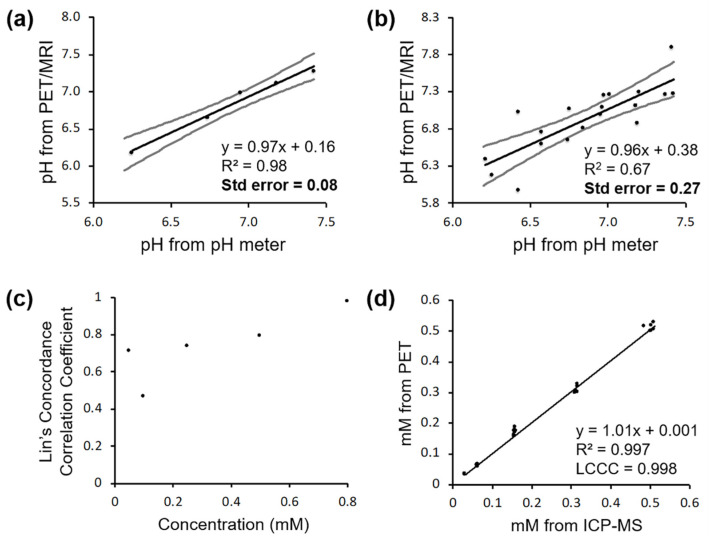
PET/MRI co-agents can measure pH in solution. (**a**) pH measured by a calibrated pH meter versus pH measured by PET/MRI in samples at a 0.8 mM concentration of MRI co-agent gives a standard error of 0.08 pH units. (**b**) pH measured by a calibrated pH meter versus pH measured by PET/MRI in samples including 0.8, 0.5, and 0.25 mM concentrations of MRI co-agent gives a standard error of 0.27 pH units. (**c**) Lin’s Concordance Correlation Coefficients were calculated for each set of samples at different MRI co-agent concentrations, and LCCCs improved as concentration increased. (**d**) MRI co-agent concentration measured by PET/MRI versus MRI co-agent concentration measured by ICP-MS shows high precision (R^2^ = 0.997) and high accuracy (LCCC = 0.998).

Similar to the samples for the pH-relaxivity calibration curve, the MRI acquisition method with shorter experimental TR times was required for samples of higher MRI co-agent concentration (0.8 mM), while samples with lower concentrations could be analyzed with longer experimental TR times. The PET/MRI co-agents were able to accurately measure pH in solution, but the precision was dependent on the concentration of the MRI co-agent. At the highest concentration of MRI co-agent (0.8 mM), a standard error of 0.08 pH units was achieved, compared to measurements from a calibrated pH meter (Figure 3a). When lower concentrations of the MRI co-agent were included (0.8, 0.5, and 0.25 mM), the standard error of pH measurements increased to 0.27 pH units (Figure 3b). An analysis using Lin’s Concordance Correlation Coefficient showed that the pH measurements from PET/MRI and a calibrated pH meter were highly correlated at higher MRI co-agent concentrations (Figure 3c). This pH measurement methodology used experimental PET results coupled with the known ratio of the co-agents in each sample to estimate the concentration of the MRI contrast agent. This concentration measurement from PET/MRI matched the MRI agent concentration determined by ICP-MS, with high precision (R^2^ = 0.997) and high accuracy (LCCC = 0.998; Figure 3d). Therefore, the errors in pH measurements by PET/MRI (Figure 3a,b) are most likely, due to more inaccurate MRI relaxation rate measurements than inaccurate estimates of MRI agent concentrations.

### 3.4. Imaging In Vivo Tumor pHe with PET/MRI

**Figure 4:**
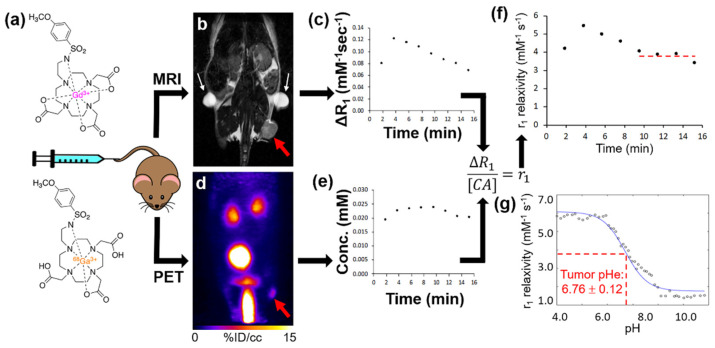
PET/MRI measures in vivo tumor pHe. (**a**) Simultaneous PET/MRI was performed with a single intravenous injection of the PET and MRI co-agents into a subcutaneous flank tumor model of MIA PaCa-2 pancreatic cancer. (**b**) Prior to injection, a baseline anatomical MR image and T_1_ map were obtained. The tumor is highlighted with a red arrow. Fiducial markers are highlighted with white arrows. (**c**) A DCE MRI scan was then started, and after 1 min a known ratio of co-agents were simultaneously injected i.v. The change in relaxation rate (ΔR_1_) was determined from the MR images. (**d**) A dynamic PET scan was simultaneously obtained with the DCE MRI scan post-injection. (**e**) The dynamic concentration of the MRI co-agent was determined from the PET images via the known injected ratio of the two agents. (**f**) The ΔR_1_ and concentration values were used to calculate r_1_ relaxivities in two-minute time frames during the dynamic PET/MRI scan. (**g**) Once the curve reached a steady value after 8 min post-injection, the average r_1_ relaxivity was compared with the pH-relaxivity calibration (same as Figure 2c) to estimate in vivo tumor pHe.

In a proof-of-concept study, a mouse with a subcutaneous tumor of MIA PaCa-2 pancreatic cancer was scanned using simultaneous PET/MRI with the MRI co-agent and the ^68^Ga version of the PET co-agent (Figure 4a). PET and MR image registration facilitated the identification of the tumor in the PET images (Figure 4b,d). The dynamic changes in ΔR_1_ relaxation rate (Figure 4c) and PET-derived MRI co-agent concentration (Figure 4e) were monitored at 7.6-sec and 2-min temporal resolutions, respectively, as averaged for the tumor region in the image. Fortunately, ΔR_1_ is linearly dependent on the concentration of the MRI co-agent (Equation (3)), so that the fast measurements of ΔR_1_ could be binned in 2-min intervals without concerns of improper weighting (a non-linear dependence would raise concerns about improper weighting in this binning process). The r_1_ relaxivity was determined from ΔR_1_ and concentration from each two-minute interval, and converted to pHe based on the pH-relaxivity calibration (Figure 2c). This pHe value was found to be consistent for the four measurements, made eight min after injection. The average of these four pHe measurements after eight min post-injection was 6.76 ± 0.12 pH units. This pH value agreed with previous studies of the subcutaneous flank model of MIA PaCa-2 pancreatic cancer [25,26], and other small animal models of human cancers that have moderate tumor metabolism [4,8,10,27,28].

Our simultaneous PET/MRI methodology was used to image two additional mice with a subcutaneous flank MIA PaCa-2 tumor, using the ^64^Cu-based PET co-agent along with the MRI co-agent (Figure S10a). Unfortunately, the PET images showed poor tumor uptake and high liver uptake of the ^64^Cu radionuclide. The low PET detection in the tumor was insufficient to estimate tumor pHe. This observation was made with both mice, indicating that this poor tumor uptake ^64^Cu radionuclide was reproducible.

### 3.5. Stability and Metabolism Studies of PET Co-Agent

**Figure 5:**
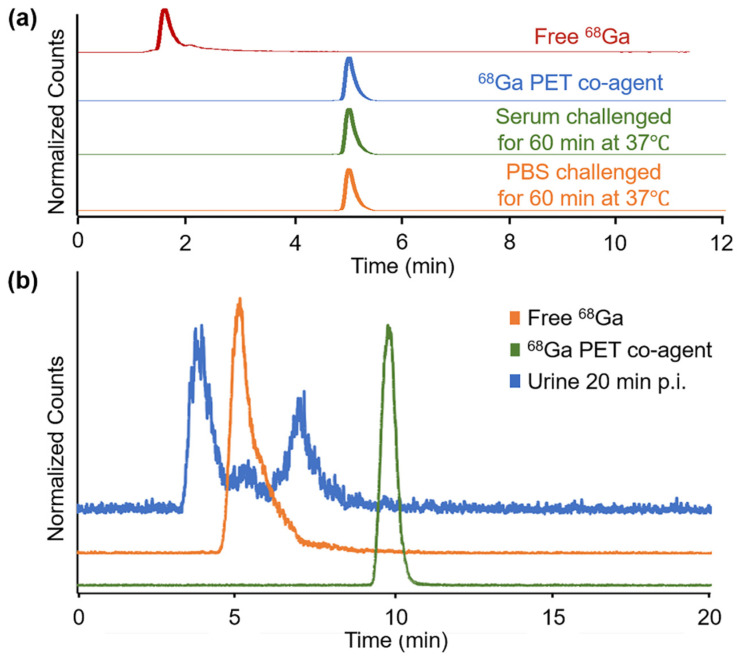
Stability and metabolism of the ^68^Ga PET co-agent. (**a**) RadioHPLC of the ^68^Ga PET co-agent (blue trace) was incubated in human serum (green trace) and PBS (orange trace) for one hour at 37 °C and showed complete stability, with no presence of free ^68^Ga (red trace). (**b**) RadioHPLC of the ^68^Ga PET co-agent from urine collected 20 min post-injection from the mouse imaged with simultaneous PET/MRI, as shown in Figure 4. The results showed the presence of two new species (blue trace) that were different than intact ^68^Ga PET co-agent (green trace) or free ^68^Ga (orange trace).

Stability studies of the ^68^Ga PET co-agent in PBS and human serum showed complete stability of the agent for up to 1 h (Figure 5a). The ^64^Cu PET co-agent also demonstrated complete stability in PBS and human serum for up to 1 h (Figure S10). In addition, the ^64^Cu PET co-agent was challenged in the presence of the MRI co-agent, to determine if the chemical equilibrium of the MRI co-agent led to the dechelation of the Cu ion. The compound was found to be completely stable in the presence of the MRI co-agent (Figure S10).

The urine of the mice injected with the PET/MRI co-agents showed no presence of the intact PET co-agents (Figure 5b and Figure S11). For the ^68^Ga PET co-agent, two species were identified with radioHPLC, and neither species matched the intact agent or free ^68^Ga (Figure 5b). For the ^64^Cu PET co-agent, the only species present in the urine was free ^64^Cu, indicating the de-chelation or transchelation of this ^64^Cu-based agent in vivo (Figure S11b). This result was consistent with our in vivo PET/MRI results with the ^64^Cu PET co-agent, which showed low uptake of the ^64^Cu radionuclide in the tumor, which is expected if de-chelation or transchelation occurs.

## 4. Discussion

We developed the synthesis of the MRI co-agent, with an overall yield of 59%, which improved on the previously reported synthesis of this agent, which had an overall yield of 17% [21]. All compounds including the DOTA ligand framework required purification by HPLC due to the basicity of the nitrogen atoms in the ligand, and their interaction with acidic normal phase silica. The purification of the final chelated MRI co-agent proved difficult due to high acid sensitivity, preventing the use of TFA in the HPLC solvent system. We found that the use of lower concentrations of formic acid led to the minimal dechelation of the complex during purification. Chelations of the radiometals were performed in high radiochemical yields, with short reaction times and high radiochemical purity. The low concentration of ^68^Ga in the eluent from the generator was acceptable for the small-scale reactions in our studies. However, a large-scale production in the future will benefit from the pre-concentration of the ^68^Ga eluent [29], or the use of lower volume cyclotron-produced ^68^Ga [30].

A large excess of the precursor was used in the radiochemical reactions, and was not removed during C18 cartridge purification. However, only the ratio of PET radioactivity (in units of μCi) to MRI concentration (in units of mM) was necessary for our pH measurements, so that this precursor did not affect our results. Moreover, if our approach with PET/MRI co-agents was used to detect a molecular biomarker such as a cell receptor, the excess precursor, as well as the high concentration of MRI co-agent, would out-compete the radiolabeled PET co-agent for the molecular target, essentially becoming a classic “blocking study” of a targeting PET agent [31]. Fortunately, the pHe of the tumor microenvironment is an environmental biomarker rather than a molecular biomarker, so that the radiolabeled PET co-agent does not compete with excess precursor or MRI co-agent to exist in this tumor microenvironment. Therefore, measuring tumor pHe is an outstanding application for our PET/MRI co-agent approach.

A major assumption of our PET/MRI approach is that the PET and MRI co-agents are delivered to the tumor in the same ratio as in the injection volume. Our use of ^68^Ga was a logical approach for designing the PET co-agent, because the ^68^Ga-based PET co-agent has the same charge as the Gd-based MRI co-agent, and the DOTA derivative is identical for the PET and MRI co-agents. Therefore, the PET and MRI co-agents should have the same pharmacokinetic delivery to tumors [32,33,34]. Our in vitro tests in PBS and human serum showed that both PET co-agents were stable, which supported our assumption. However, each PET co-agent demonstrated instability in vivo, raising concerns that a loss of the metal ion will change the charge of the complex, and invalidate the assumption of identical pharmacokinetics of the PET and MRI co-agents. ^64^Cu is known to de-chelate from DOTA, and can be easily transchelated by enzymes in the liver, which often leads to high retention of the metal ion in the liver [35]. This was observed in our in vivo PET/MR images with the ^64^Cu PET co-agent (Figure 5a), and was validated by the radioHPLC of extracted urine post-injection (Figure 5b). Therefore, a ^64^Cu-based PET agent was an unsuccessful choice for our PET/MRI co-agent approach.

Interestingly, radioHPLC from urine at 20 min p.i. from mice injected with the ^68^Ga PET co-agent showed two polar species that are not free ^68^Ga, suggesting in vivo metabolism of the DOTA derivative in this PET co-agent, rather than de-chelation (Figure 4b). Despite this evidence for degradation, the PET/MRI co-agents using ^68^Ga were able to measure tumor pHe during a 16-min dynamic PET/MRI scan. This result suggests that the PET co-agent may be stable within the tumor extracellular microenvironment, and only metabolized during excretion from the body. Therefore, a ^68^Ga-based PET agent was a useful choice for our initial in vivo PET/MRI approach.

Similar to the development of other biosensors and pharmaceutical agents, our next phase of development could include the refinement of the PET/MRI co-agents to reduce in vivo metabolism and increase biocompatibility. For example, additional ligands may be introduced to the co-agents to improve their hydrophilicity. These improvements would directly lead to safety and toxicity tests that will be required for eventual approval for clinical use. In addition, our in vivo tests of the ^68^Ga PET co-agent used human serum, while our in vivo tests exposed this agent to mouse serum. While this difference in sera may be inconsequential, future in vitro tests to refine the PET/MRI co-agents should use mouse serum to be consistent with in vivo pre-clinical studies.

The PET/MRI co-agents accurately measured pH in solution, although the precision of this pH measurement was dependent on the concentration of the MRI co-agent (Figure 3a,b). Different applications of pHe measurements can accommodate different standard errors in pH measurements (Figure 1b). For example, tumors typically have a pHe that is substantially lower than normal tissue, inflammation, and infection [3,7,8]. A PET/MRI measurement of pHe with a lower co-agent concentration that has a standard error of 0.27 units (Figure 3b) would be acceptable for distinguishing tumor versus these other tissue types. Similarly, malignant tumors can have high glycolytic metabolism relative to benign tumors, which may cause a large difference in pHe that can accommodate lower diagnostic precision [36]. However, tumor pHe changes during early response to therapy are closer to 0.1–0.2 pH units [10,11], so that higher concentrations of the MRI co-agent will be needed to accurately detect early therapeutic effects. Importantly, our results indicated that even moderate errors in ^68^Ga radioactivity measurements were still able to precisely measure MRI co-agent concentrations (Figure 3d). Therefore, we attribute the errors in pH measurements to the MRI process of our approach. Higher concentrations of the MRI co-agent and more accurate ways to measure T_1_ times will lead to improvements in pH estimates with simultaneous PET/MRI.

Our in vivo studies demonstrated that PET/MRI co-agents can be used to measure pHe in the microenvironment of a subcutaneous flank tumor model of pancreatic cancer following 6 weeks of tumor growth to reach a 5 mm diameter. This demonstration can be expanded in the future to evaluate other tumor models, including models of other cancer types; models with orthotopically implanted tumors or spontaneously forming tumors; and tumors with different stages of growth and volume. For these future studies, a solid tumor must have a patent vascular system that allows for the sufficient uptake of the co-agents for detection with MRI and PET. For example, severely necrotic tumors that have little or no patent vasculature would not be good candidates for evaluation with PET/MRI co-agents. However, measurements of tumor acidosis are more relevant for metabolically active tumors than for severely necrotic tumors, which mitigates this limitation.

## 5. Conclusions

Radiometal-based PET/MRI co-agents were synthesized in high yields and purity. The r_1_ relaxivity of the MRI co-agent depended on pH, the ΔR_1_ increase in relaxation rate caused by the MRI co-agent could be measured with MRI, the PET radioactivity measurements could accurately estimate the concentration of the MRI co-agent, and these results could be combined to accurately measure pH, with a standard error as low as 0.08 pH units. The ^64^Cu PET co-agent experienced transchelation in vivo, and was not able to measure tumor pHe using PET/MRI. Despite evidence that the ^68^Ga PET co-agent degraded in urine after 20 min, this PET co-agent and the MRI co-agent were used to measure in vivo tumor pHe within 16 min. This initial study demonstrated a feasible workflow for using PET/MRI co-agents to measure tumor pHe, and indicated future improvements for simultaneous PET/MRI.

## Data Availability

Data are contained within the article and are available by contacting Mark D. Pagel, mdpagel@mdanderson.org.

## Supplementary Materials

The following are available online at https://www.mdpi.com/article/10.3390/bios12020134/s1, Figure S1: NMR of compound **1**, Figure S2: NMR of compound **2**, Figure S3: NMR of compound **3**, Figure S4: NMR of compound **4**, Figure S5: LRMS of compound **1**, Figure S6: LRMS of compound **2**, Figure S7: LRMS of compound **3**, Figure S8: LRMS of compound **4**, Figure S9: LRMS of compound **5**, Figure S10: RadioHPLC trace of ^64^Cu PET co-agent when challenged in solution, Figure S11: Metabolism of the ^64^Cu PET co-agent.
