# Supplementary material for: Radiometal-Based PET/MRI Contrast Agents for Sensing Tumor Extracellular pH
Source: Biosensors (Basel). 2022 Feb 20;12(2):134. doi: 10.3390/bios12020134 (PMC8870419; doi:10.3390/bios12020134)
Supplement: Supplementary file 1 [file biosensors-12-00134-s001.zip › biosensors-1543613-supplementary.pdf]

biosensors

Supplementary Materials

Radiometal-Based PET/MRI Contrast Agents for Sensing Tumor Extracellular pH

Alyssa C. Pollard [1,2], Jorge de la Cerda [2], F. William Schuler [2], Tyler R. Pollard [3], Aikaterini Kotrotsou [2], Federica Pisaneschi [2] and Mark D. Pagel [2,*]

[1] Department of Chemistry, Rice University, Houston, TX 77005, USA; acp4@rice.edu
[2] Department of Cancer Systems Imaging, MD Anderson Cancer Center, Houston, TX 77054, USA; jorge.delacerda@mdanderson.org (J.d.l.C.); fschuler@mdanderson.org (F.W.S.); aikaterini.kotrotsou@gmail.com (A.K.); fpisaneschi@mdanderson.org (F.P.)
[3] Department of Chemistry and Biochemistry, Georgia Institute of Technology, Atlanta, GA 30332, USA; polko987@outlook.com
* Correspondence: mdpagel@mdanderson.org; Tel.: +001-(713)-205-8515

1. NMR Spectra of Synthesized Compounds

Citation: Pollard, A.C.; de la Cerda, J.; Schuler, W.; Pollard, T.R.; Kotrotsou, A.; Pisaneschi, F.; Pagel, M.D. Radiometal-Based PET/MRI Contrast Agents for Sensing Tumor Extracellular pH. *Biosensors* **2022**, *12*, 134. https://doi.org/10.3390/bios12020134

Received: 22 December 2021
Accepted: 16 February 2022
Published: 20 February 2022

Publisher's Note: MDPI stays neutral with regard to jurisdictional claims in published maps and institutional affiliations.

Copyright: © 2022 by the authors. Licensee MDPI, Basel, Switzerland. This article is an open access article distributed under the terms and conditions of the Creative Commons Attribution (CC BY) license (https://creativecommons.org/licenses/by/4.0/).

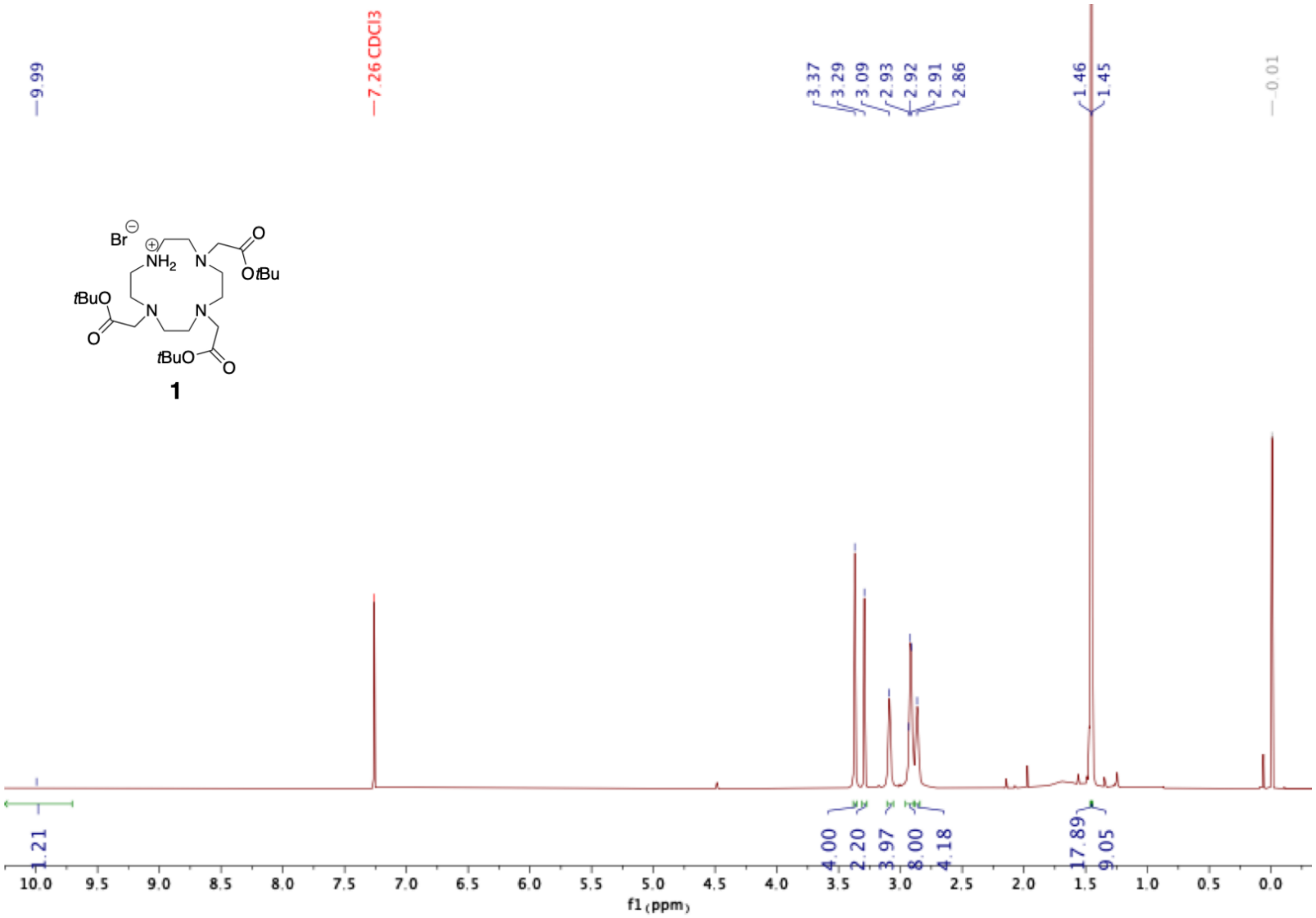

Figure S1. NMR spectrum of compound **1.**

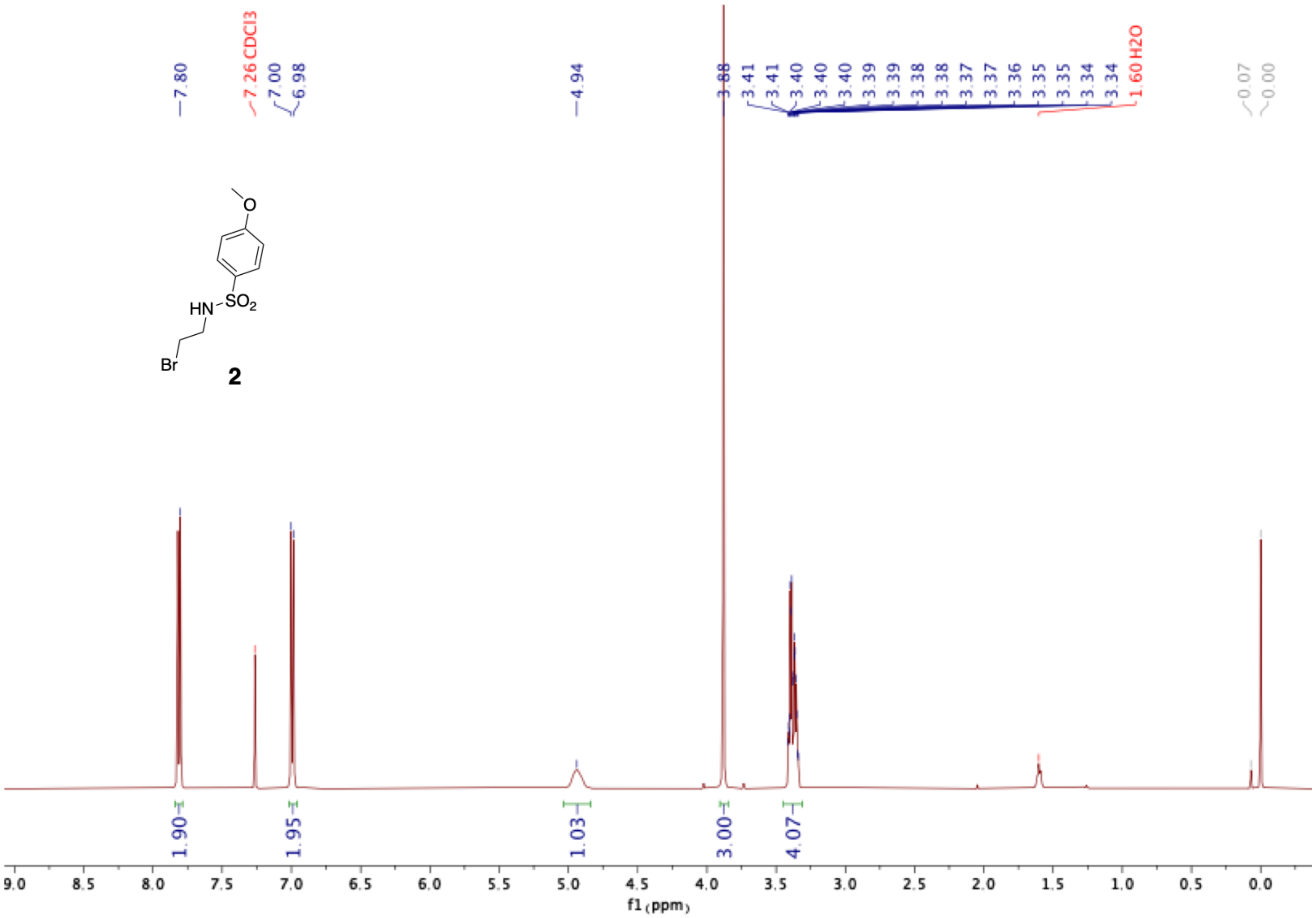

Figure S2. NMR of compound **2.**

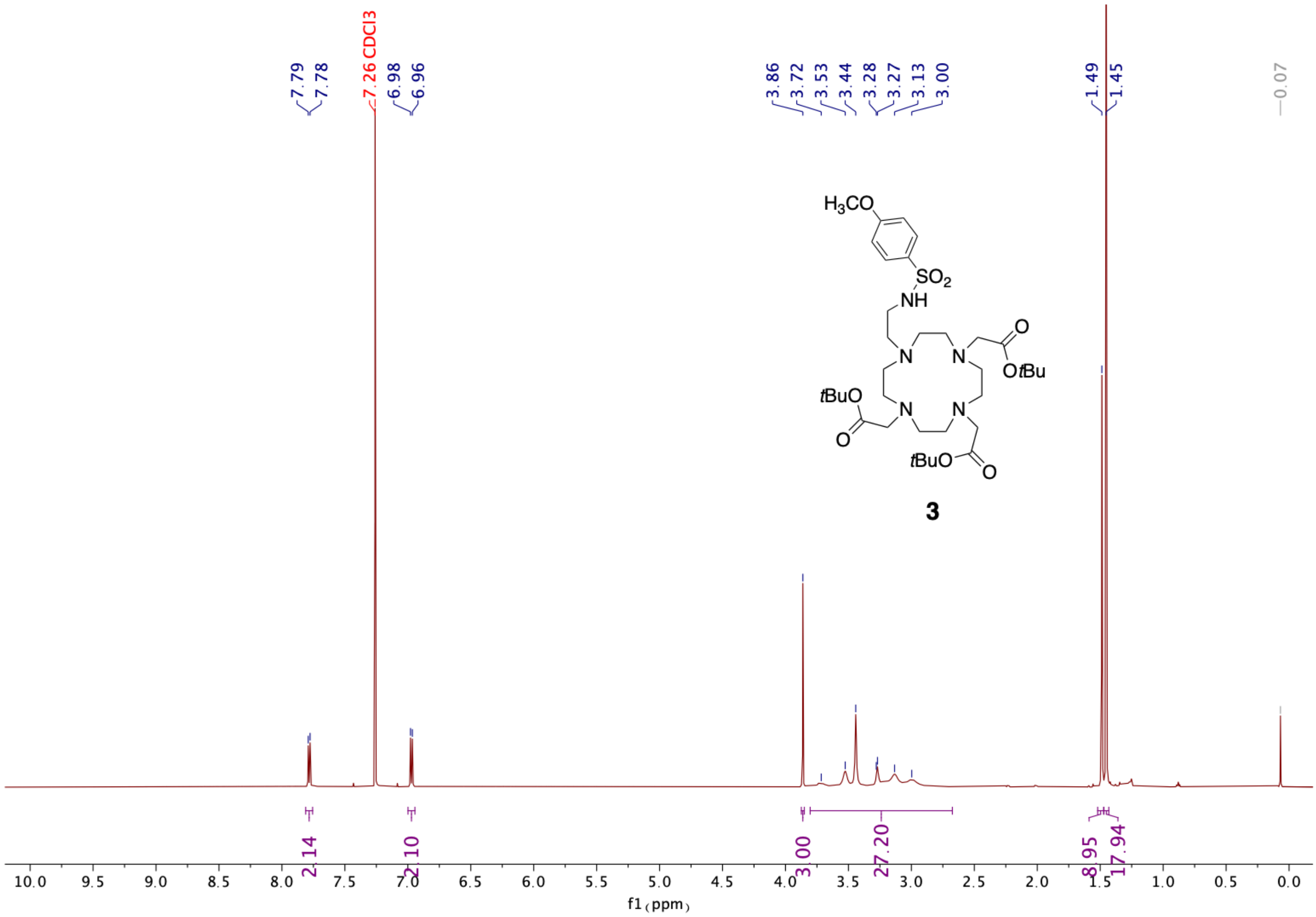

Figure S3. NMR of compound **3.**

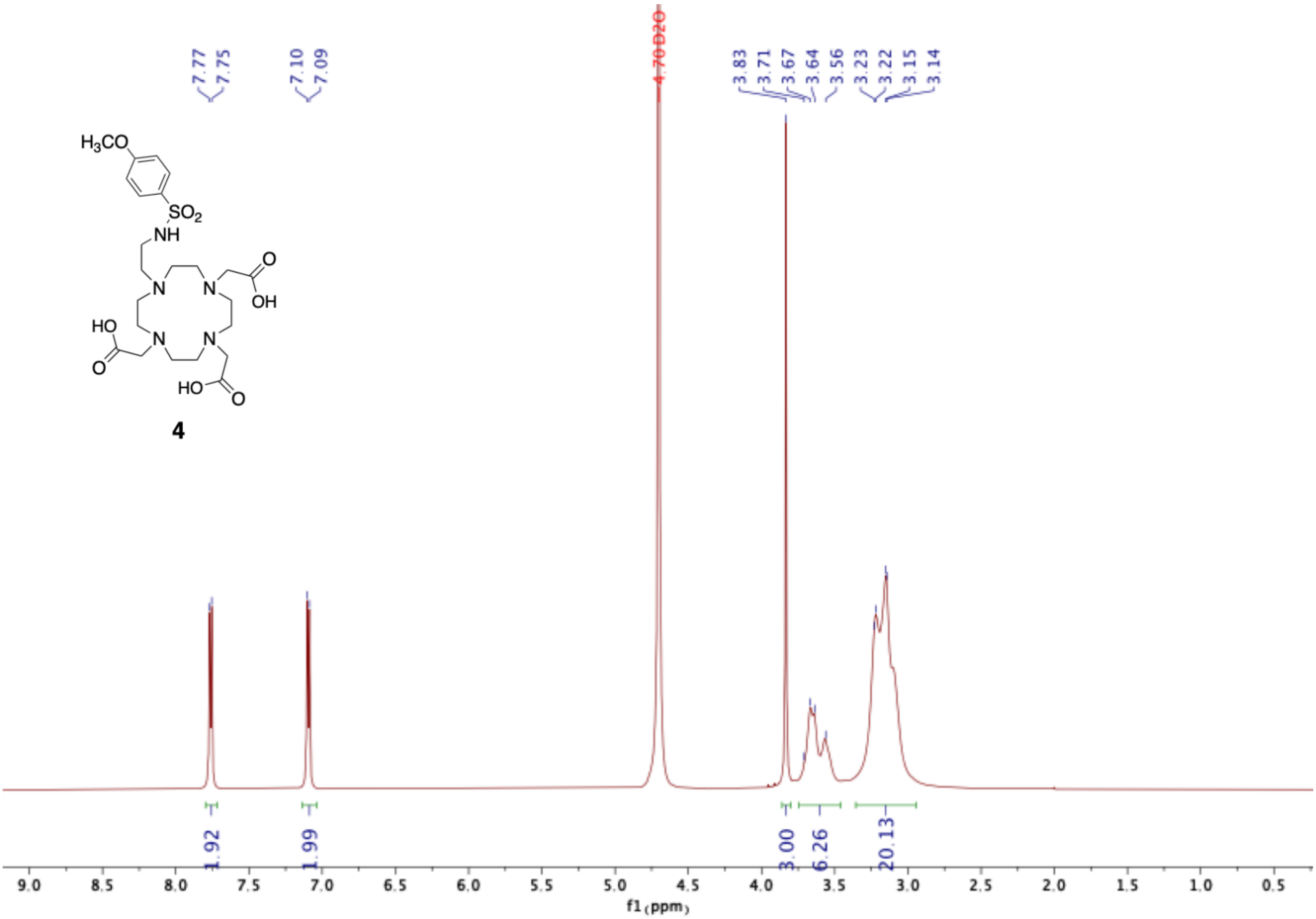

Figure S4. NMR of compound **4.**

2. LRMS of Synthesized Compounds

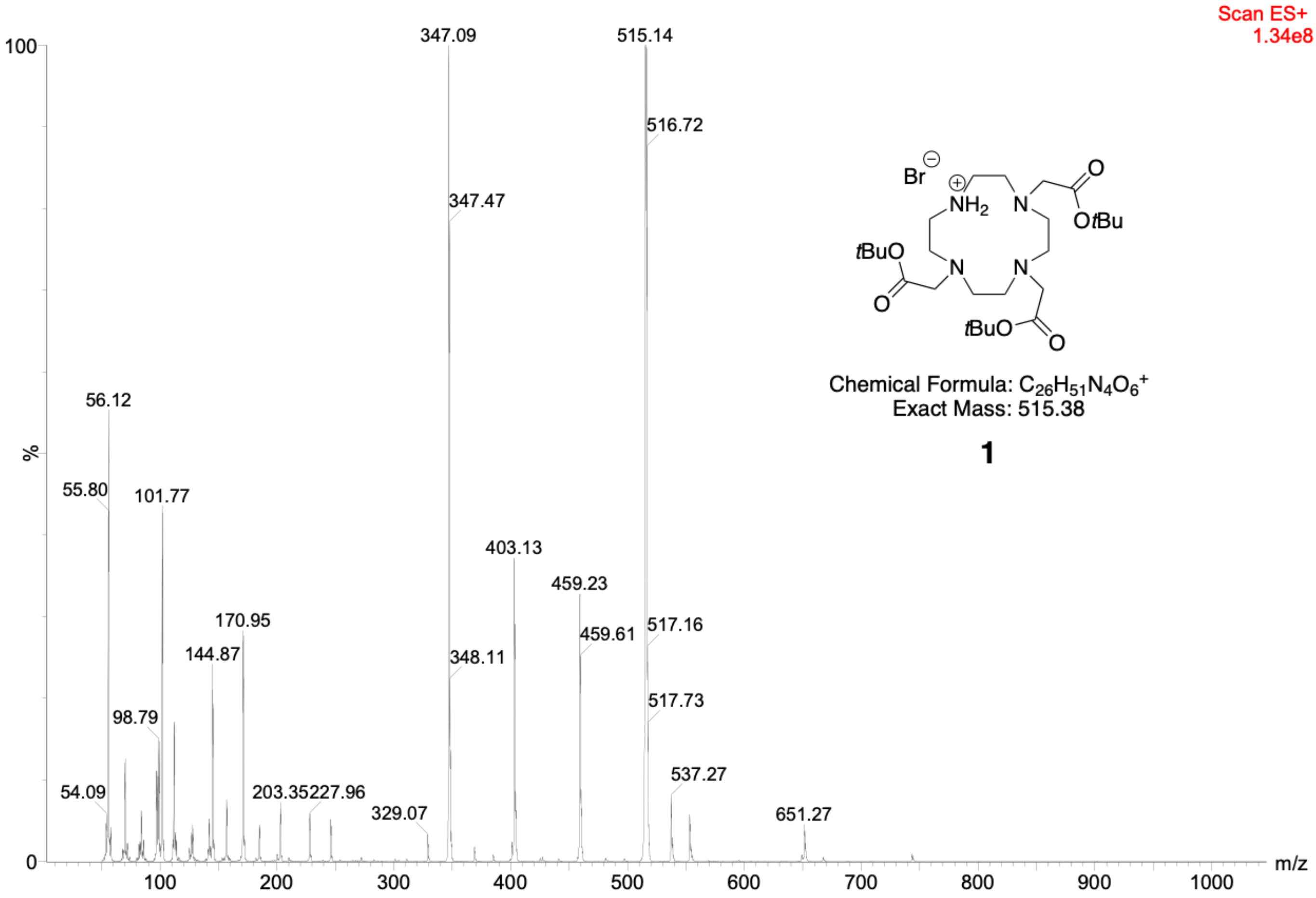

Figure S5. LRMS of compound **1.**

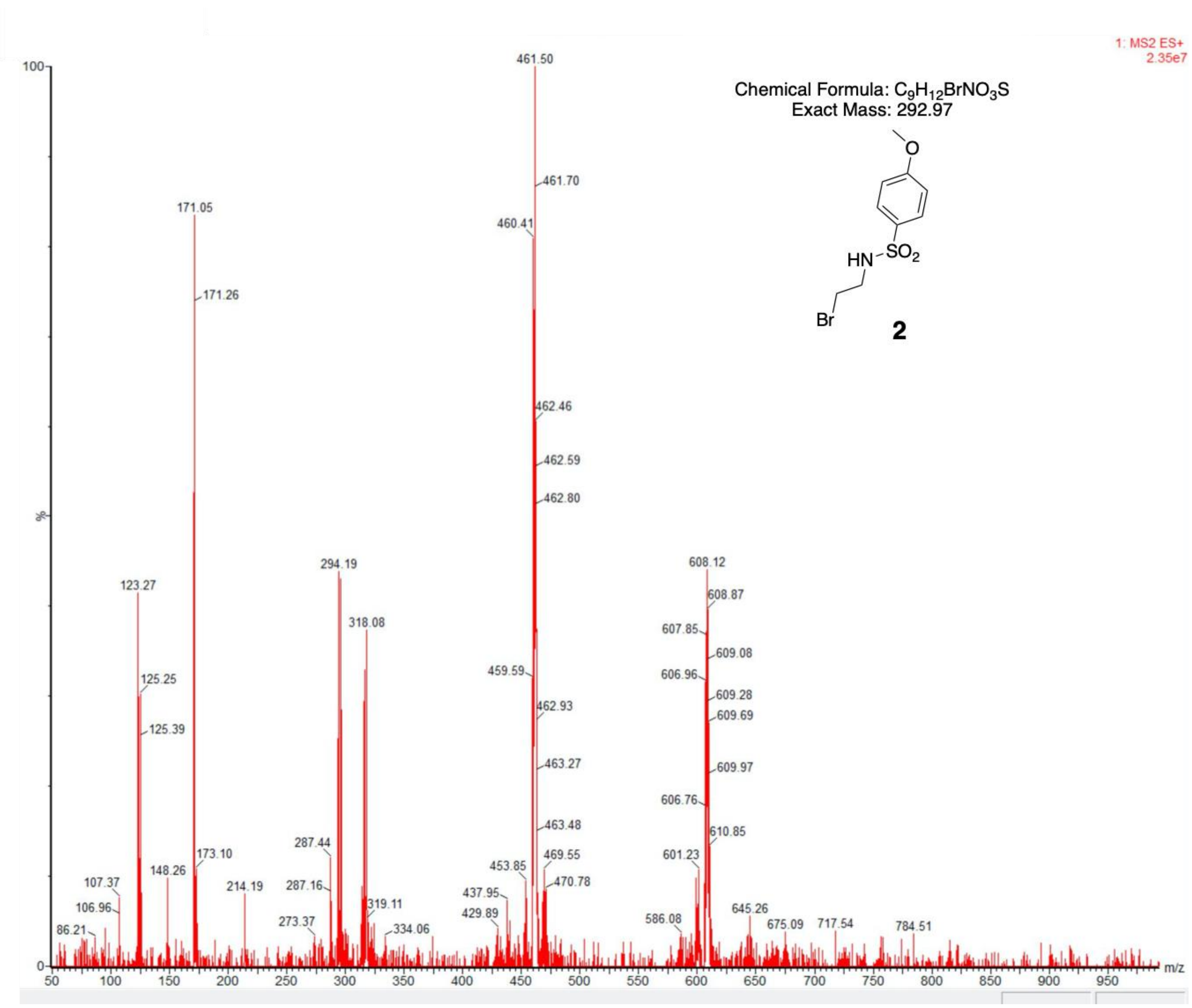

Figure S6. LRMS of compound **2.**

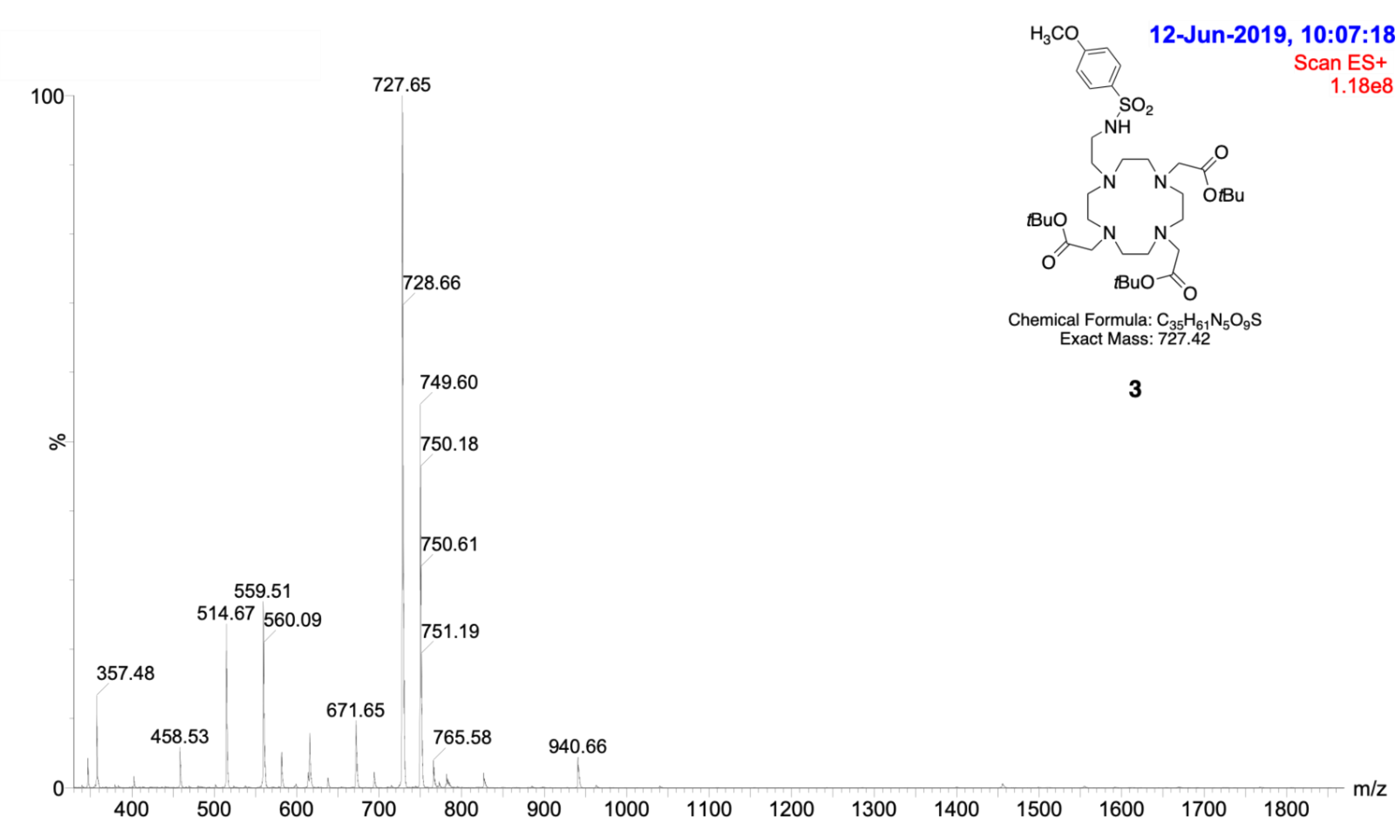

Figure S7. LRMS of compound **3.**

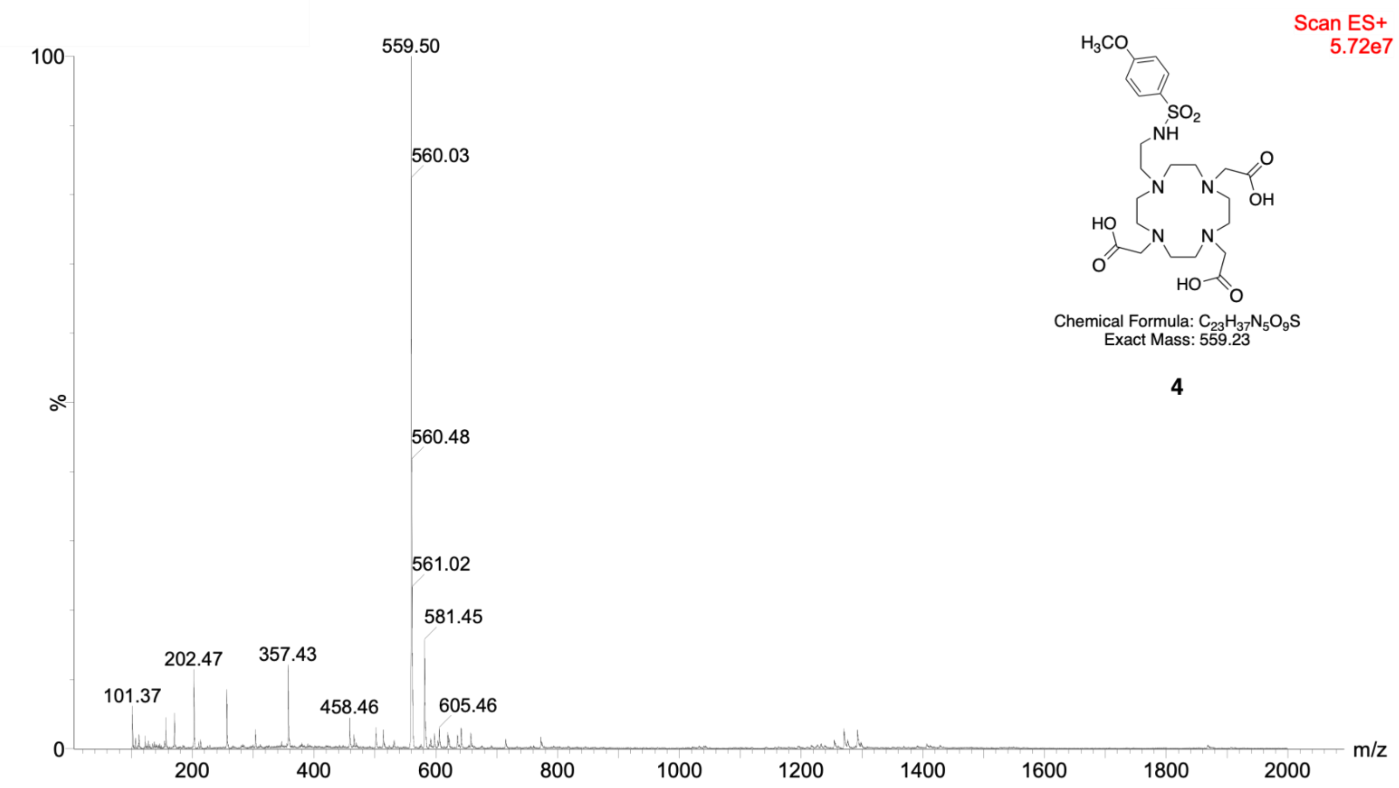

Figure S8. LRMS of compound **4.**

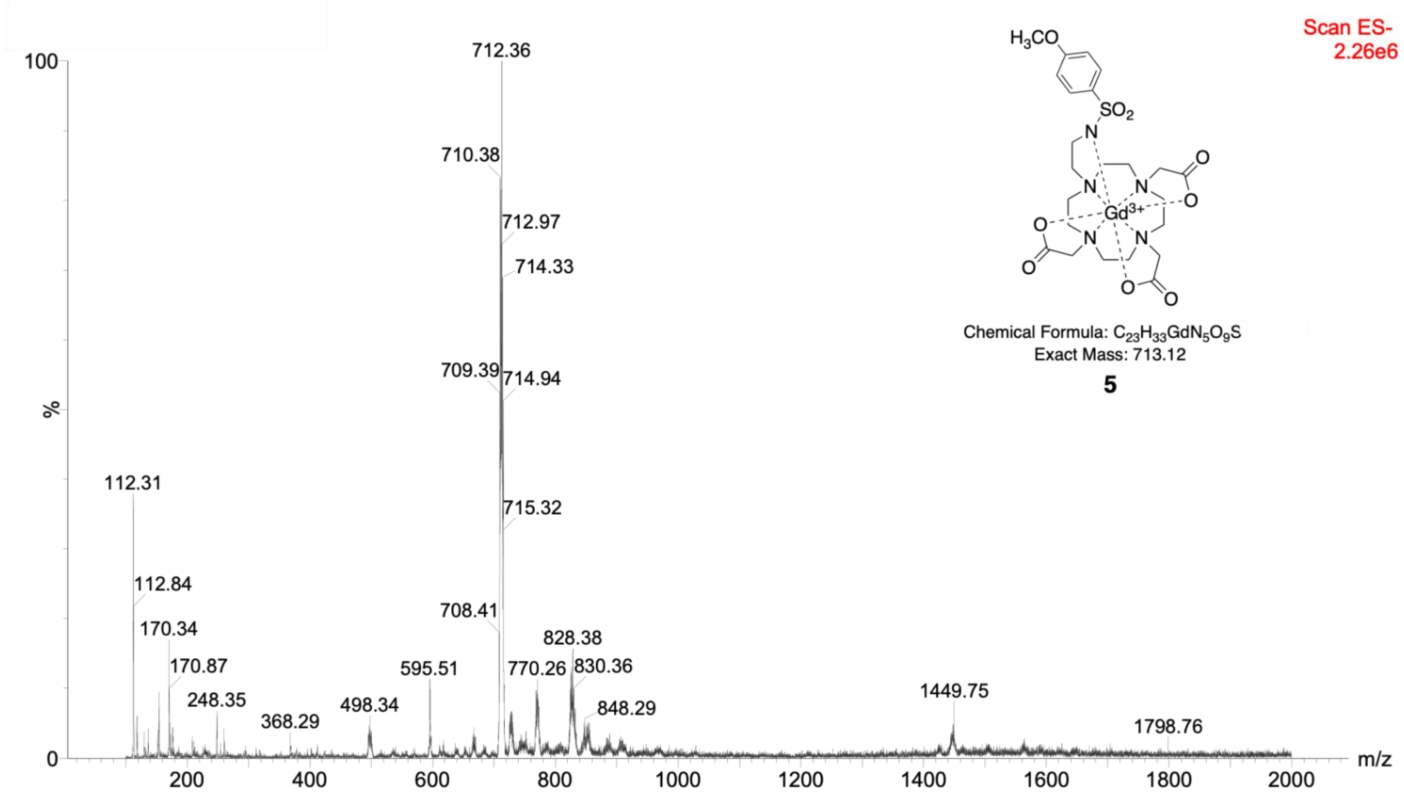

Figure S9. LRMS of compound **5.**

3. RadioHPLC Traces of PET Co-Agents

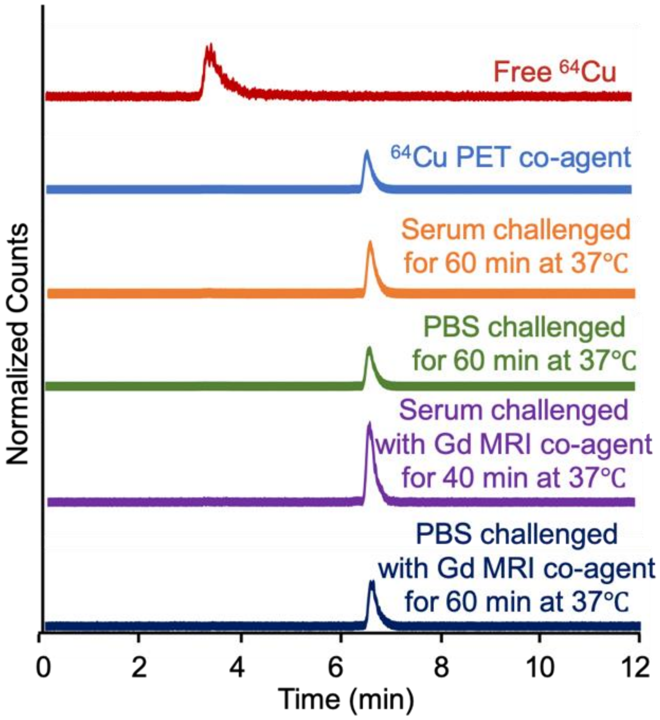

Figure S10. *RadioHPLC trace of ^{64}Cu PET co-agent when challenged in solution.* The ^{64}Cu PET co-agent (blue trace) was incubated in human serum (orange trace) and PBS (green trace) for one hour at 37ºC and showed complete stability with no presence of free ^{64}Cu (red trace). The ^{64}Cu PET co-agent was also incubated in the presence of the MRI co-agent in serum (purple trace) and PBS (dark blue trace) and showed complete stability.

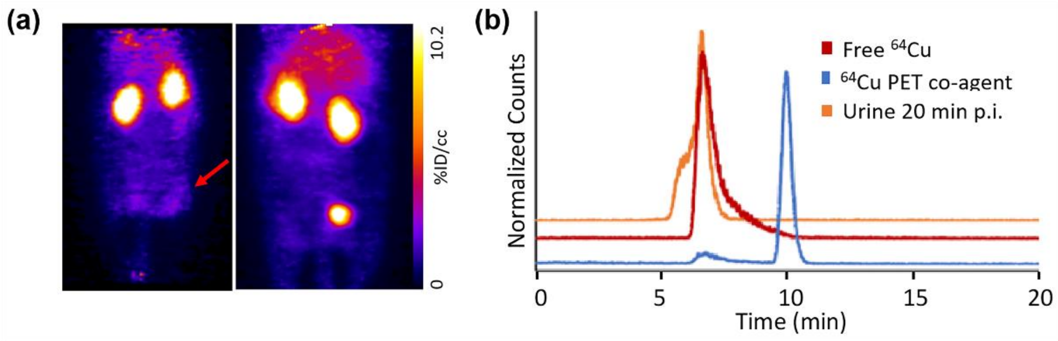

Figure S11. *Metabolism of the ^{64}Cu PET co-agent*. (**a**) A PET image from the dynamic PET/MRI scan using the ^{64}Cu PET co-agent showed poor tumor uptake and high liver uptake. (**b**) RadioHPLC of urine collected from the mouse after the dynamic PET/MRI scan (orange trace) showed the presence of only free ^{64}Cu (red trace) rather than showing the intact ^{64}Cu PET co-agent (blue trace), indicating *in vivo* de-chelation of the ^{64}Cu PET co-agent.
